# Vaccination after Exposure to Small Pox

**Published:** 1846-05-01

**Authors:** Samuel Salisbury


*Vaccination after exposure to Small-pox.* By Samuel Salisbury, Jr. M. D. From the Boston Medical and Surgical Journal.—Mrs. S——, a married woman, 36 years of age, unprotected by vaccination, left home to visit a friend who resided in the city of Rochester, eighteen miles distant. She found her friend confined to her bed, and affected by natural small-pox. After sitting by her bed-side about half an hour, she crossed the street to a physician's office, was vaccinated, and returned home. The progress of the vaccine disease was regular, the vesicle forming perfectly, and attended with considerable constitutional fever on the eighth and ninth days. Virus was taken from the vesicle on the eighth day, and inserted in the arms of seven persons, five of whom were children, by a physician who was ignorant of the exposure of this woman to the contagion of small-pox. On the eighteenth day after her visit to Rochester, I was called, and found her in the eruptive period of small-pox, symptoms of which she told me had manifested themselves four days before. The disease was of great severity, its violence being in no degree mitigated by the prophylactic power of the recent vaccine disease. Of the children vaccinated from her arm, three had the cow-pock succeeded by modified or mild small-pox, but partial desquamation having taken place when I visited them on the twenty-first day from the vaccination. In the other two, the vaccine vesicle was fully perfected, and not succeeded by any symptoms of small-pox. The two adults, who were vaccinated with virus taken from the woman's arm, left the place, and I learned nothing further of them, than the fact that they both escaped the small-pox. Neither of the five children had been vaccinated previously, and circumstances did not favour the idea of a direct communication of the disease from the woman to them.

The foregoing case leads us to the following conclusions.

I. Vaccination on the day of exposure to the contagion of small-pox is not always a protection. Might it not have proved a protection, had it been deferred for four or five days, so that the manifestation of the constitutional symptoms of both diseases would have been synchronous?

II. It is unsafe to make use of vaccine virus taken from persons who are in a location where small-pox exists; whether the small-pox in these children be viewed as caused by the vaccination, communicated through the medium of the physician, or as arising from an epidemic influence.

A consideration of the general principles which are supposed to govern the operation of morbid poisons, and exanthematous diseases, as well as known facts, impress us with a sense of the importance of revaccination with virus which has been renewed by reproduction from its original source, the cow:

I. As a test of individual idiocrasy;

II. In order to determine the perfection of the virus previously introduced;

III. Lest all the symptoms of the cow-pock, both constitutional and local, were not produced by the previous vaccination.

*Avon, N. Y., March 3d*, 1846.

In connection with the foregoing, the following cases of vaccination after exposure to small-pox, from our own experience, may perhaps possess interest for some of our readers.

A patient was removed, after the eruption had fully appeared, from a room in which there was an infant, some four or six months old, which had never been vaccinated. The following day it was vaccinated. Three days afterward it was ascertained that the vaccination would prove unsuccessful, and it was revaccinated, which was successful at the usual time, and the child escaped the small-pox. This infant had been in the habit of lying on the same bed with the patent, receiving caresses, &c., up to the time when the patient was removed from the house to the small-pox hospital.

The second case is as follows: a young man with Rubeola was removed from the jail to the small-pox hospital, and occupied a room contiguous to that in which a patient was lying ill with the small-pox, constant communication existing between the two rooms. He was supposed to have the small-pox when he was removed. The day after his admission he was vaccinated. The vaccination was unusually long in producing its effects, so that there was reason to fear it would prove unsuccessful. At length, however, specific pustules were produced, and the patient escaped the small-pox. This patient had never before been vaccinated.

These cases illustrate the efficacy of vaccination as a protective after full exposure to the contagion of small-pox.

